# A folded π-system with supramolecularly oriented dipoles: single-component piezoelectric relaxor with NLO activity†

**DOI:** 10.1039/d2sc06141d

**Published:** 2023-01-04

**Authors:** Soumi De, Deepak Asthana, Chinthakuntla Thirmal, Sudhir K. Keshri, Ram Krishna Ghosh, Geeta Hundal, Raju Kumar, Satyendra Singh, Ratnamala Chatterjee, Pritam Mukhopadhyay

**Affiliations:** a School of Physical Sciences, Jawaharlal Nehru University New Delhi – 110067 India m_pritam@mail.jnu.ac.in; b Department of Chemistry, Ashoka University Sonipat Haryana 131029 India; c Department of Physics, Indian Institute of Technology Delhi New Delhi – 110016 India; d Department of Electronics & Communications Engineering, Indraprastha Institute of Information Technology Delhi New Delhi 110020 India; e Department of Chemistry, Guru Nanak Dev University Amritsar Punjab-143005 India; f Special Centre for Nanoscience, Jawaharlal Nehru University New Delhi – 110067 India; g VNR Vignana Jyothi Institute of Engineering and Technology Hyderabad Telangana 500 090 India

## Abstract

Organic molecules with an active dipole moment have a natural propensity to align in an antiparallel fashion in the solid state, resulting in zero macroscopic polarization. This primary limitation makes the material unresponsive to switching with electric fields, mechanical forces, and to intense laser light. A single-component organic material that bestows macroscopic dipole-driven electro-mechanical and optical functions, *e.g.*, piezoelectric, ferroelectric and nonlinear optical (NLO) activity, is unprecedented due to the design challenges imparted by crystal symmetry and dipole orientations. Herein we report a crystalline organic material that self-assembles with a polar order (*P*_1_), and is endowed with a high piezoelectric coefficient (*d*_33_–47 pm V^−1^), as well as ferroelectric and Debye-type relaxor properties. In addition, it shows second harmonic generation (SHG) activity, which is more than five times that of the benchmark potassium dihydrogen phosphate. Piezoelectric force microscopy (PFM) images validated electro-mechanical deformations. Piezoresponse force spectroscopy (PFS) studies confirmed a signature butterfly-like amplitude and a phase loop. To the best of our knowledge, this is the first report of a folded supramolecular π-system that manifests unidirectionally oriented dipoles and exhibits piezoelectricity, ferroelectricity, and has excellent ability to generate second harmonic light. These findings can herald new design possibilities based on folded architectures to explore opto-, electro- and mechano-responsive multifaceted functions.

## Abstract 2

We have shown that organic folded structures can be utilized to elicit piezo-, ferroelectric and efficient second harmonic generation – all within a single component, macroscopically dipole active system.

## Graphical abstract

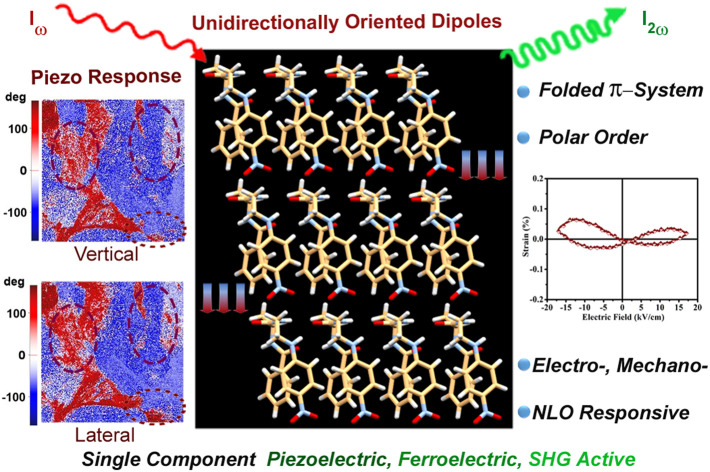

Understanding the structural and mechanical behaviour of bio-molecules and polymers under a physiological electric field provides insight for designing dipole-active organic materials. The complexity of the hierarchy and folding of biomolecular structures greatly controls their physical properties. Dipolar molecular materials with oriented dipoles can confer nonlinear optical (NLO) activity, as well as piezo and ferroelectricity.^1^ Synthetic aromatic oligoamides are one of the potential candidates for mimicking the folded structure of biopolymers.^2^ A molecule is driven into a folded geometry depending upon the flexibility and bulkiness of its backbone, H-bonding proficiency, π–π stacking interactions, and local dielectric environments.^3^ Therefore, it has been suggested to utilize non-covalent interactions in designing novel organic materials to unravel functions that are founded on certain symmetry-restricted properties and oriented dipoles.^4^

The ease of tuning the solubility, low temperature processability, and lesser toxicity compared to inorganic materials are key advantages to strive towards new organic systems with multifaceted electro-mechanical and NLO response.^5^ This has been nicely illustrated by the groups of Tokura, Stupp, Reddy, Ghosh and others.^1,4,5,14,15^ Recent reports by the groups of Xiong, Boomishankar, Yang and others majorly focus on pioneering systems based on inorganic and hybrid materials.^6^ These are mostly based on two-component ionic systems. However, studies on switchable electrical, mechanical and NLO activity integrated within an all-organic single-component system remain in their infancy.

We envisaged that flexible built-in π-conjugated folded conformations can enable a system to function like a molecular spring by enhancing low-energy controlled reversible mechanical deformations.^7–9^ Along with this, introducing a chiral backbone in the dipolar molecule is anticipated to attain the desired electro-mechanical and NLO functions, which are restricted by the crystal symmetry and hierarchical ordering of the molecules.

In this work, we have successfully tested this hypothesis using a simplistic design approach. Our design involves two aromatic π-units: π_a_ bearing a carbonyl functionality and π_b_ comprising an elementary polarizable *p*-nitroaniline unit, in addition to a flexible chain and l-alanine amino acid as the chirality-inducing element that acts as a chiral spacer (Scheme 1). Intriguingly, molecule 1 forms a polar, highly oriented dipolar assembly, as validated from its single-crystal X-ray structure. Further, piezoresponse force microscopy (PFM) and piezoresponse force spectroscopy (PFS) studies confirmed piezoelectricity in the nano-regime with a high converse piezoelectric coefficient (*d*_33_). Polarization *vs.* electric field (*P*–*E*) loop measurements corroborated ferroelectric properties. Dielectric measurements of this material revealed Debye-type relaxor properties. Furthermore, the noncentrosymmetric crystalline platform and the supramolecularly oriented dipoles led to high second harmonic generation (SHG) activity. To the best of our knowledge, this is the first report of an organic single-component material with a dipolar folded architecture that is electro-mechanically responsive as well as SHG active.

**Scheme 1:**
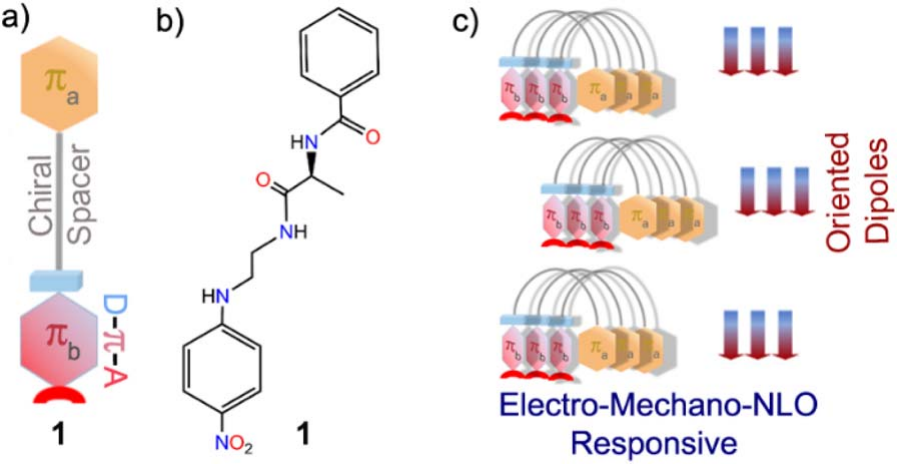
(a) Schematic representation of molecule 1 with its important structural components: aromatic unit-π_a_, dipolar unit-π_b_ and the chiral spacer; (b) molecular structure of 1; (c) supramolecular assembly of 1 to form highly oriented dipoles to function as an electro-mechano and NLO responsive single-component material.

The synthesis of compound 1 was achieved in three steps (see ESI† for details). Firstly, *N*′-(4-nitrophenyl)ethane-1,2-diamine (1a) was synthesized by reacting 4-nitrochlorobenzene with ethylenediamine, and was used in excess in the presence of CuCl_2_. Next, 2-benzamidopropanoic acid (1b) was synthesized by reacting benzoyl chloride with l-alanine in a basic medium. Finally, 1a and 1b were coupled together using DCC (*N*,*N*′-dicyclohexylcarbodiimide) and HOBt (1-hydroxybenzotriazole) to form the desired peptide linkage in 1. Compound 1 was purified by column chromatography and isolated in 45% yield.

Manifestation of piezoelectricity and ferroelectricity as well as NLO activity from a crystalline system involves satisfying stringent crystallographic symmetry constraints. Gratifyingly, we grew good quality single crystals by slow evaporation of a solution of 1 in ethanol. Single-crystal X-ray crystallographic data validated a triclinic polar space group *P*_1_, which signifies the absence of any rotation axes (other than the identity rotation axis of order 1), rotary-inversion axes, screw axes, or planes.^10^

Interestingly, 1 adopts a folded or an ‘inverted U’ shape in the crystal (Fig. 1a). The crystal packing is governed by two sets of mutually perpendicular H-bonds along the *a* and *c* axes. There are two distinct interlayer H-bonds along the *c* axis, between the –N=O⋯H–C– of the next layer backbone (2.485 Å) and the aromatic = C–H⋯O=C– (2.529 Å) along the *c* axis (Fig. 1b), which are longer than the intermolecular H-bonded array along the *a* axis. There is another set of three H-bonds along the *a* axis, corresponding to O⋯H distances of 2.072 Å, 2.219 Å, and 2.255 Å (Fig. 1c). These interactions in 1 assist it to adopt a folded structure and a unidirectional molecular arrangement in the crystal. As a result of such a folded structure, the planes containing the two π-units do not interact facially as they form an angle of 69.4° (Fig. 1d).

**Fig. 1:**
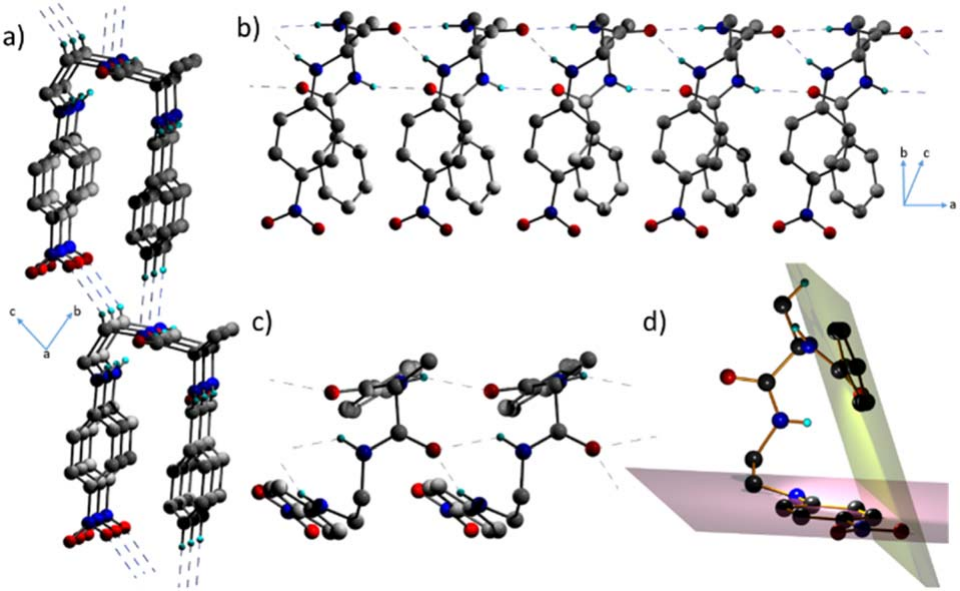
POV-ray representation of the single-crystal structure of 1 showing: (a) its folded structure and ability to form highly oriented dipoles utilizing H-bonding interactions; (b) and (c) various intermolecular non-covalent H-bonding interactions; (d) angle subtended by the two π-units. The atoms C, O, N and H are represented in grey, red, blue and light cyan, respectively.

This implies that the π clouds of the two aromatic rings weakly interact with each other and thus allow flexibility of the folded architecture. Notably, such a parallel orientation of dipoles in π-conjugated folded structures is rare. Therefore, control over H-bonds can be useful in designing dipole active supramolecular structures.^11^ Since 1 crystallized in a polar non-centrosymmetric space group with supramolecularly oriented dipoles, manifestation of switchable electrical, mechanical and nonlinear optical properties was anticipated.^12^

To examine the piezoelectric properties, we performed PFM alongside topographical studies of crystalline thin films of 1. The morphology of the thin films was studied by AFM and scanning electron microscopy, which revealed fibrous structures (Fig. S4a and b†). Fig. S4† shows the topography of the crystalline surface and its corresponding powder XRD pattern. PFS of the nano-domains revealed a butterfly-shaped amplitude loop and a phase loop of nearly 180° phase difference both within the long range (Fig. 2a and b) and a narrow range of DC bias voltage (Fig. S5†). The converse piezoelectric coefficient, *d*_33_, of 1 was determined as ∼46.7 pm V^−1^ (ESI, eqn S1†). The *d*_33_ value was averaged over the values obtained at four different points.

**Fig. 2:**
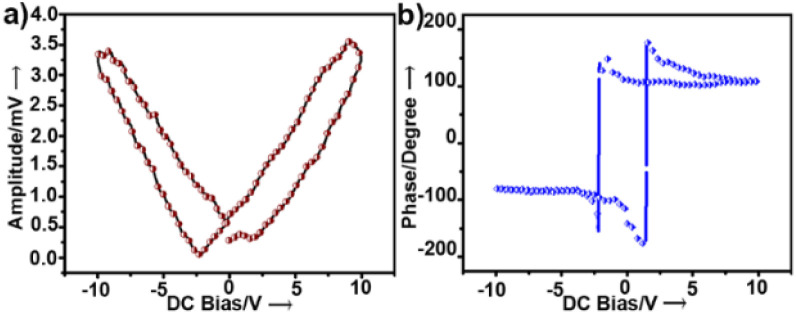
(a) Amplitude and (b) phase hysteresis loops of a thin film of 1 recorded by PFS as a function of DC bias voltage applied for 1 s.

From the PFM studies, the piezo-responses were phase and amplitude images collected at room temperature. Green to pale-yellow contrast in the amplitude images signified the extent of electro-mechanical deformation vertically and horizontally (Fig. 3a and b). Brighter yellow regions in the lateral amplitude image suggested favourable lateral deformation over vertical. Fig. 3c and d highlight the macromolecular insight of the phase of oriented dipoles in the thin film. The blue to red color contrast implies anti-parallel to parallel orientation of the polar-axis induced by the applied electric field. We attempted to record domain switching images at reverse voltages to address the ferroelectric behaviour. However, a thin film of 1 did not show reversible switching characteristics within ± 15 V. Consequently, crystalline thin film surface imaging helped to understand the responsible vectorial component for brighter lateral amplitude.

**Fig. 3:**
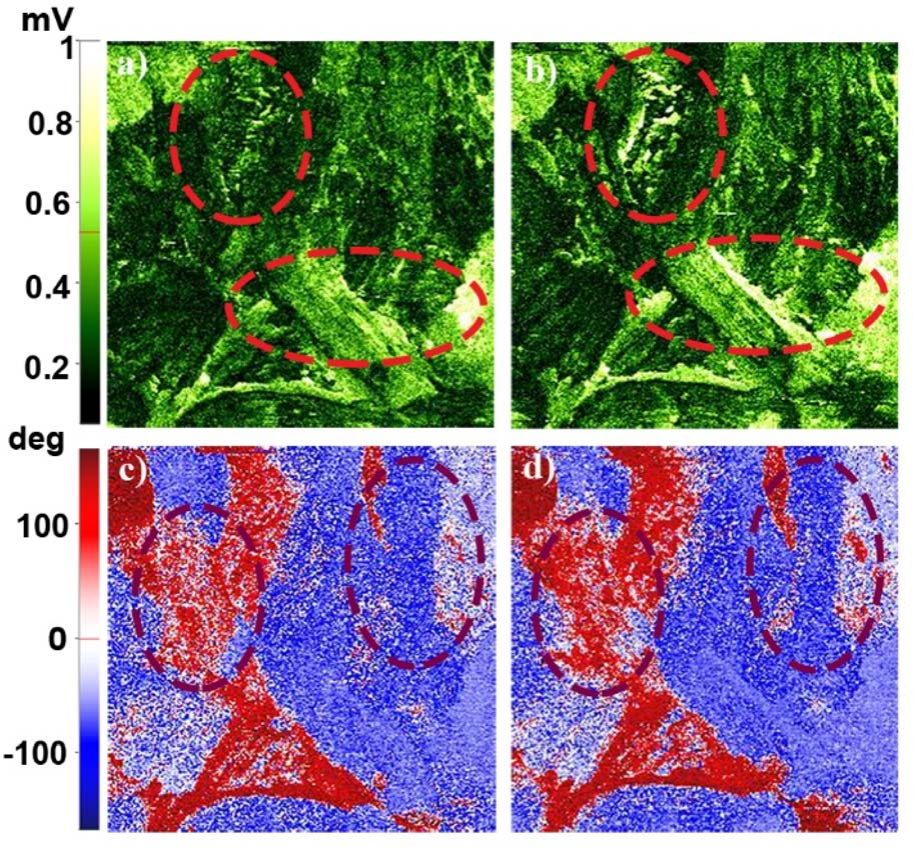
PFM amplitude and phase images of a thin film of 1 on a 5 × 5 μm^2^ area; amplitude images of the (a) vertical and (b) lateral directions; and phase images of the (c) vertical and (d) lateral piezo-response.

Further, vertical and lateral amplitude PFM images were collected in mutually perpendicular *X* and *Y* scanning directions (Fig. 4). In the *X*-scanning, defined as ‘00’, the cantilever long-axis is perpendicular to the scan direction, whereas in the *Y*-direction, defined as ‘90′, the cantilever long-axis is parallel to the scan direction. If the dipoles are oriented perpendicular (out-of-plane) to the *X*–*Y* surface plane, then the tip always deflects vertically in both the *X* and *Y* scans in vertical mode as induced by the vertical oscillation of the dipoles. Here, in the vertical mode of the *X*- and *Y*-scanning images (Fig. 4a–b), the most occurring amplitude values are nearly the same, but the maximum number of pixels are nearly two times more in *Y* scanning than for *X* scanning. This indicates that the vertical deflection of the tip is not purely coupled with the out-of-plane oscillation.

**Fig. 4:**
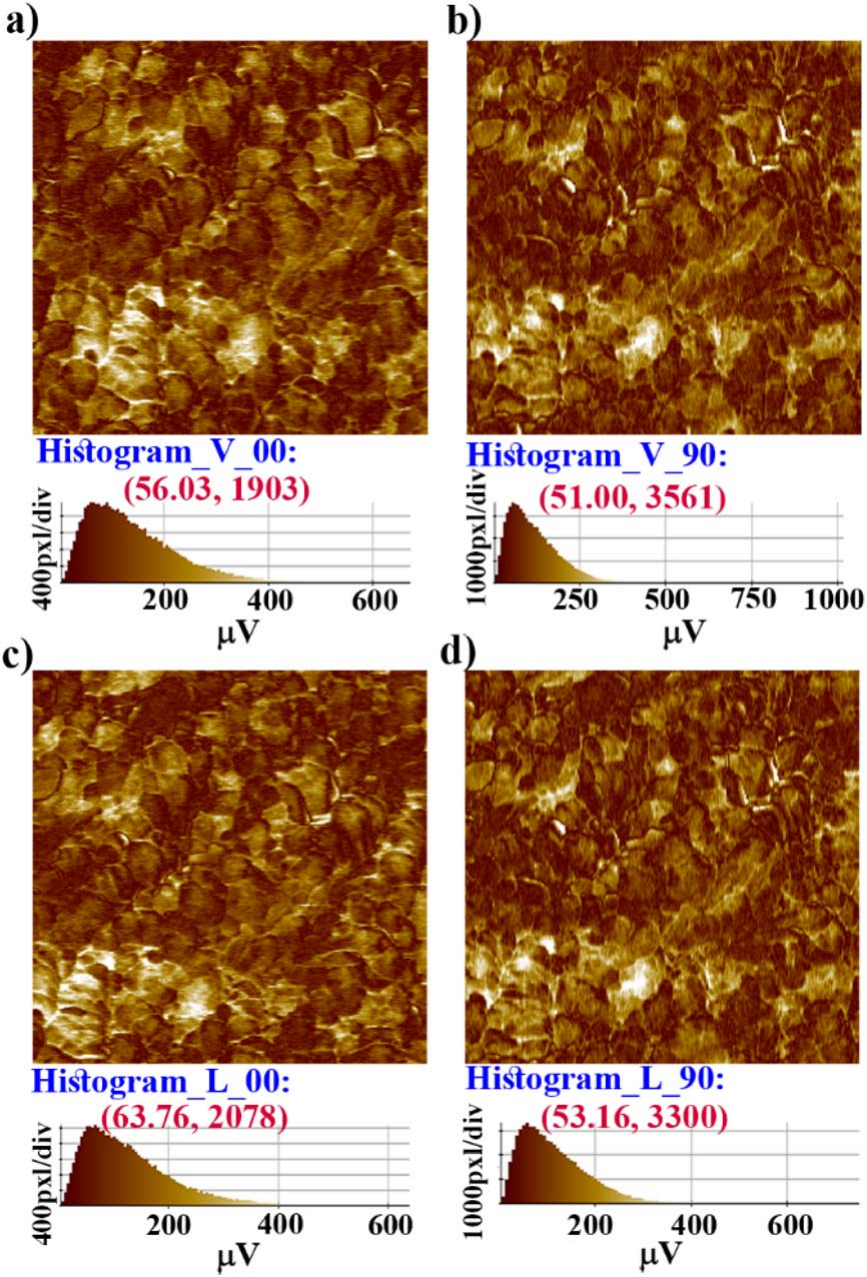
PFM amplitude image of a crystalline thin film of 1: (a) and (b) vertical and (c) and (d) lateral amplitude images collected by scanning the *X* and *Y* directions; the indication of 0 and 90 is relative. The corresponding histograms show that the maximum number of pixels have the most occurring amplitude value of deformation (in μV).

While the dipoles are aligned in parallel to the long-axis of the cantilever, fully or partially, the vertical deflection of the cantilever gets affected due to the buckling motion of the tip and appeared in vertical imaging.^13^ However, lateral mode images produced the most occurring deflection amplitude (64 μV) in the *X* scan and maximum pixels (3300) in the *Y* scan with comparable amplitude value (Fig. 4c–d). These results suggest that better electro-mechanical coupling occurs in the *X*–*Y* plane of the crystalline surface of 1. In addition, bulk piezoelectric studies on a crystalline pellet of 1 at 300 K revealed characteristic strain *vs. E* loops, confirming the feature of a piezoelectric material (Fig. 5a).

**Fig. 5:**
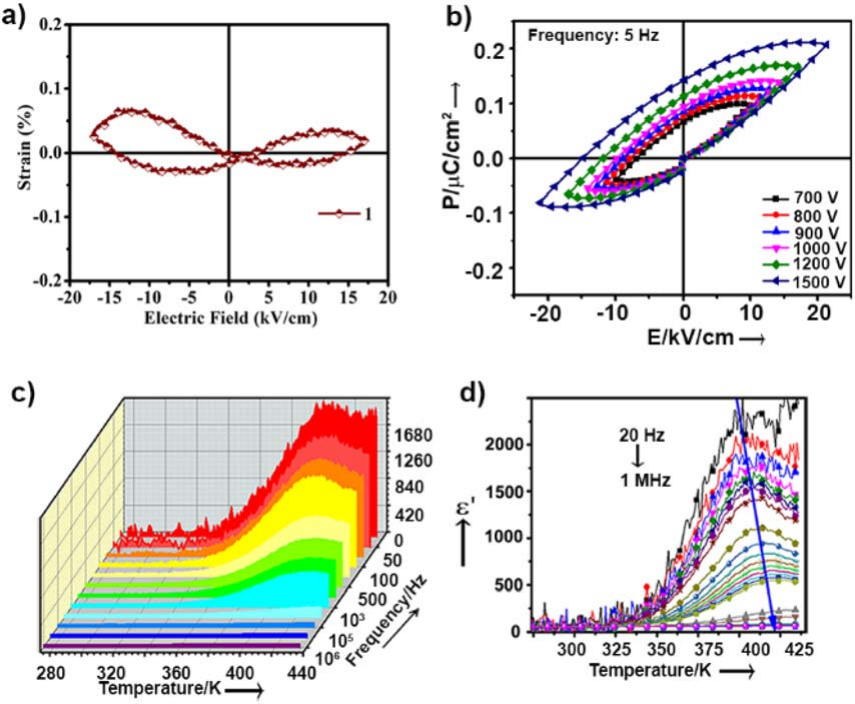
(a) Strain-electric field loops at AC voltage amplitude, 1200 V, 5 Hz frequency at 300 K recorded in pellet form (0.6 mm diameter and ∼0.7 mm thick) by a precision materials analyzer (radiant technologies). (b) *P*–*E* loop of 1 under various oscillatory electric field amplitudes. (c) Dielectric constant (real part, *ε*′) as a function of temperature and frequency at an oscillatory voltage 0.5 V. (d) The blue arrow shows the dispersion of the hump of the dielectric constant towards a higher temperature.

We calculated the spontaneous polarization in the bulk along the three-axes by using a vd-DF2 functional in the Berry phase method. The computed values of the crystal lattice parameters were in good agreement with the experimental ones (ESI, Table S5†). We obtained nearly equal values of polarization, *i.e.*, 32 and 30 along the *b*-and *c*-axes, respectively, and 20 along the *a*-axis. Therefore, these calculated results validated that 1 has significantly high polarization for piezo-response in all directions. Comparatively weak and long H-bonds along the *c*-axis are easily polarizable in an applied electric field than the shorter H-bonds along the *a*-axis. This explanation is in line with the theoretically predicted polarization value along the *c* axis and the analysis of the PFM amplitude images.

Ordered networks of donor–acceptor type systems have been shown to be potential candidates towards all-organic ferroelectric materials.^14^ It is noteworthy that hydrogen bonded molecules undergo reversible polarisation switching that enables the system to exhibit ferroelectric properties and can be an effective strategy to create organic ferroelectric analogues of potassium dihydrogen phosphate (KDP).^15^ For ferroelectricity to transpire, it is crucial to have aligned dipoles in these H-bonded systems.^16^ In our case, single-crystal X-ray data of molecule 1 revealed a fully oriented molecular arrangement in the bulk, and a polar space group, which led us to examine its ferroelectric properties. *P*–*E* loop measurements were performed in pellet form at 300 K. Distinct saturation of polarization in both sides of the *P*–*E* hysteresis loops was not observed (Fig. 5b). The gradual increase in AC voltage amplitude at a constant frequency retained the shape of the asymmetric loop. The asymmetric nature in the *P*–*E* and the strain *vs.* applied field loops suggest non-ideal or leaky ferroelectric characteristics for 1.

One of the reasons for the asymmetric *P*–*E* loop could be the intrinsic characteristic of the folded dipoles. As revealed from the single-crystal XRD studies, 1 exhibits weak intramolecular π-interactions that hold the molecules in the folded form and the intermolecular H-bonds hold the dipoles in an organized, dipole-oriented fashion. Therefore, improper reorientation of the initially oriented dipoles can occur while applying the reverse electric field, resulting in an asymmetric loop. The other possible reason could be the evolution of defects generated during the sample preparation or difference in charge trapping in the crystalline interface between the two electrode regions during the application of external fields, which builds up an internal field, leading to an asymmetric loop. This aspect has been seen in other ferroelectric materials.^17^ We measured the leakage current of 1 and found it to make a negligible contribution as the magnitude of current remained very low, in the order of ∼10^−7^ (Fig. S6†).

Unlike normal ferroelectrics that show a sharp transition temperature, a round-shaped hump is observed in the temperature (273 K to 433 K) and frequency dependent dielectric constant (real part, *ε*′) measurements of 1 (Fig. 5c). This hump further shifts towards a higher temperature and gradually gets flattened with increasing frequency (Fig. 5d). These are characteristics of Debye-type dielectric relaxors, whereby dipoles are not able to reorient under the reverse applied field, and thus fail to exhibit a sharp transition at high frequency. Fig. S7† shows that the dielectric dispersion at different frequencies obeyed the modified Curie–Weiss law [ESI eqn S1†]. The slope of this plot determines the diffusion constant (*γ*) value to be ∼1.7, which further suggests that 1 has relaxor properties.^18^

Finally, we performed SHG measurements on a crystalline sample of 1, which showed 5.3 times SHG activity with respect to the benchmark potassium dihydrogen phosphate (KDP). The crystalline sample showed high chemical and optical stability with an input laser pulse energy of 3–5 mJ per pulse. This frequency doubling efficiency of 1 is amongst the best within the family of organic single-component systems, as recently exemplified by Seferos and co-workers.^1*d*^ The polarizable intermolecular H-bonds have better coupling with the applied field instead of an individual dipole, which results in NLO and electrical response.^19^ Beside these H-bonds, the skewed conformation between the two aromatic rings of 1 brings about flexibility in the backbone. Therefore, the vibration of local folding motifs along *b* axis remarkably adds an effect in the piezo-response.^20^

## Conclusions

In summary, we have shown for the first time that organic folded structures can be utilized to elicit piezo- and ferroelectric properties, as well as efficient second harmonic generation, all within a single-component, macroscopically dipole active system. This multi-function generation is unprecedented in a single-component all-organic system. The ability of the material to form supramolecularly oriented dipoles along with the manifestation of the polar crystalline order *P*_1_ (*i.e.*, devoid of any rotation axes, rotary-inversion axes, screw axes, or planes) results in the evolution of a multifaceted electro-, mechano- and nonlinear optical response. PFM imaging of the crystalline thin films in vertical and lateral modes enabled understanding of the orientation of the dipoles in their immobile phase. PFS studies revealed a high piezoelectric coefficient (*d*_33_–47 pm V^−1^) as well as a characteristic butterfly-like loop, as mandated by a piezoelectric material. Bulk piezoelectric studies on a crystalline pellet revealed characteristic strain *vs. E* loops, while bulk ferroelectric studies revealed characteristic *P*–*E* loops. Dielectric studies confirmed Debye-type relaxor properties. In addition, SHG activity was observed, which was more than five times that of the benchmark potassium dihydrogen phosphate. Thus, single-component supramolecular systems with special molecular architectures can be conceptualized in preference to bicomponent organic systems to elicit piezo-, electro- or other stimuli-based responses.^21^ These systems can be further engineered for exciting energy and light harvesting applications.^22^ We believe these findings will stimulate new design possibilities based on folded architectures and new dipole active π-systems^23^ to explore opto, electro and mechano-responsive multifaceted functions.

## Data availability

The datasets supporting this article have been uploaded as part of the ESI.†

## Author contributions

The molecular design was conceived by D. A. and P. M. Synthesis of the molecule was conducted by D. A. S. D carried out the PFM and PFS studies. The ferroelectric studies were carried out by C. T, S. D., and R. K. in the supervision of R. C., and S. S. G. H. helped in the crystal data collection and analysis with contributions from D. A. Computational studies were completed by S. K. K. and R. K. G. The manuscript was written by S. D. with contributions from D. A. All the authors agreed on the final version of the manuscript.

## Conflicts of interest

There are no conflicts to declare.

## Supplementary Material

SC-014-D2SC06141D-s001

SC-014-D2SC06141D-s002
