# Supplementary material for: A folded π-system with supramolecularly oriented dipoles: single-component piezoelectric relaxor with NLO activity
Source: Chem Sci. 2023 Jan 4;14(10):2547–52. doi: 10.1039/d2sc06141d (PMC9993858; doi:10.1039/d2sc06141d)
Supplement: SC-014-D2SC06141D-s001 [file SC-014-D2SC06141D-s001.pdf]

Electronic Supplementary Material (ESI) for Chemical Science.
This journal is © The Royal Society of Chemistry 2023

Supporting Information

A Folded π-system with Supramolecularly Oriented Dipoles: Single-Component Piezoelectric Relaxor with NLO-Activity

Soumi De,[a] Deepak Asthana,[b] Chinthakuntla Thirmal,[c] Sudhir K. Keshri,[a] Ram Krishna Ghosh,[d] Geeta Hundal,[e] Raju Kumar,[f] Satyendra Singh,[f] Ratnamala Chatterjee,[c] Pritam Mukhopadhyay[a]

Table of Contents

1. Experimental Details ………………………………………….. S2

2. Synthesis of **1** ………………………………………….. S4

3. FT-IR Spectra ………………………………………… S5

4. NMR Spectra …………………………………………S5

5. Single crystal XRD data and structure refinement ………………………………………… S7

6. Topography and powder XRD data of thin film of molecule **1** ………………………………………… S8

7. Amplitude and Phase Plot by PFM Spectroscopy ………………………………………… S8

8. Converse Piezocoefficient Calculation ………………………………………… S9

9. Experimental and calculated XRD parameters of crystal **1** ………………………………………… S9

10. Leakage Current measurement ………………………………………… S9

11. Modified Curie-Weiss Law plot and equation …………………………………………S10

12. References………………………………………….. S10

Experimental Details

General

All chemicals were obtained from Sigma Aldrich or Spectrochem India and were freshly distilled before use. Thin layer chromatography (TLC) was carried out on aluminium plates coated with silica gel mixed with fluorescent indicator having particle size of 25 µm and was sourced from Merck India. ^1H and ^{13}C NMR spectra were recorded on a Bruker 500 MHz spectrometer in DMSO-d_6 with TMS as standard. Infra-Red spectra were recorded in KBr pellet using Varian 3100 FT-IR instrument.

Second Harmonic Generation Measurements

The second harmonic generation activity was measured by using Spectra Physics Laser (1064 nm), model Prolab 170, having a pulse width of 10 ns and a repetition rate of 10 Hz with an input laser energy of 3-5 mJ/pulse. The optical and chemical stability of the compound was checked after the SHG experiments. The scattered light was collected at perpendicular direction. The detection system consisted of a photomultiplier tube (Hamamatsu R2059) and an Oscilloscope (Tektronix TDS3052B).

Single crystal X-ray data

Good quality single crystals were grown from EtOH solution. The reported data set for the crystal was collected by mounting the crystal with paratone oil at 100 K, on a Bruker's Apex-II CCD diffractometer using Mo Kα (λ = 0.71069 Å). The data were corrected for Lorentz and polarization effects and empirical absorption corrections were applied using SADABS from Bruker. The structure was solved by direct methods using SIR-92 and refined by full-matrix least squares refinement methods based on F^2, using SHELX-97. All non-hydrogen atoms were refined anisotropically. All hydrogen atoms were fixed geometrically with their U_{iso} values 1.2, 1.2 and 1.5 times of the phenylene, methylene and methyl carbons, respectively. All calculations were performed using Wingx package. The amide hydrogens in the structure was located from the difference Fourier synthesis and were refined isotropically with U_{iso} values 1.2 times of the carrier nitrogen atoms, with fixed distances [0.88(2) Å] where needed. **CCDC** No. for **1:** 1861614

P-E loop and strain-electric field loop measurement

For ferroelectric and field induced strain measurements, the electrodes were made on both faces of the pellets using silver paste, to make a metal-dielectric-metal capacitor structure. Polarization (P) against electric field (E) loops were measured at 300 K using Radiant ferroelectric tester (Precision Premier II-USA) at 5 Hz frequency at various fields. Field-induced strain measurements were also carried out along with P-E measurements on a photonic sensor-based strain meter (MTI-2100 Fotonic Sensor, MTI instruments, USA). During electric field-induced P-E and strain measurements, sample was kept in silicon oil (DC 704) to prevent arcing.

Scanning Electron Microscopy (SEM)

The surface morphological image of thin film of **1** was collected by *Zeiss EVO40* scanning electron microscope.

Piezoelectric Force Microscopy (PFM)

Morphology and piezoelectric property were studied by atomic force microscopy (AFM), Park NX-10 system, coupled with Piezoelectric Force Microscope (PFM) on a thin film of the compound on ITO coated glass which was cleaned thoroughly with isopropylalcohol, acetone under the sonication for ~15 mins for each solvent. 15 mM solution was prepared by dissolving the compounds in dry THF and then 20 µl of the portion of this solution was casted on ITO coated glass (1×1 cm^2) surface for making thin film by a spin coater at 200 rpm speed at room temperature. Simultaneous imaging of surface topography, piezoelectric amplitude and phase were run under PFM mode with Multi75E Cr/Pt coated conductive tip (resonant frequency 75 kHz, force constant 3N/m) using AC voltage 1.5 V of 17 kHz. The DC-bias dependent piezoelectric amplitude and phase spectroscopy were also performed with the same tip.

Theoretical Calculation

The ultra-soft plane-wave pseudopotential was used as implemented in quantum ESPRESSO software for obtaining the optimized atomic positions and lattice parameters by utilizing the single crystal XRD data as initial input. We have chosen vdW-DF2 exchange-correlation energy functional which is suggested to be suitable for organic molecules for considering van der Waals or H-bond interactions. Kinetic energy cut-off of 100 Ry and 4 × 4 × 4 *k*-point mesh values were adopted while optimizing the atomic positions and lattice parameters self consistently until the change in total energy of the system reaches to a tolerance value of 10^{-3}. The Berry-phase technique was utilized for spontaneous electric polarization calculation of the crystal. Further, we have performed DFT calculation of single molecule of **1** by B3LYP functional in 3-21G basis under Gaussian 09W package.

Synthesis of 1:

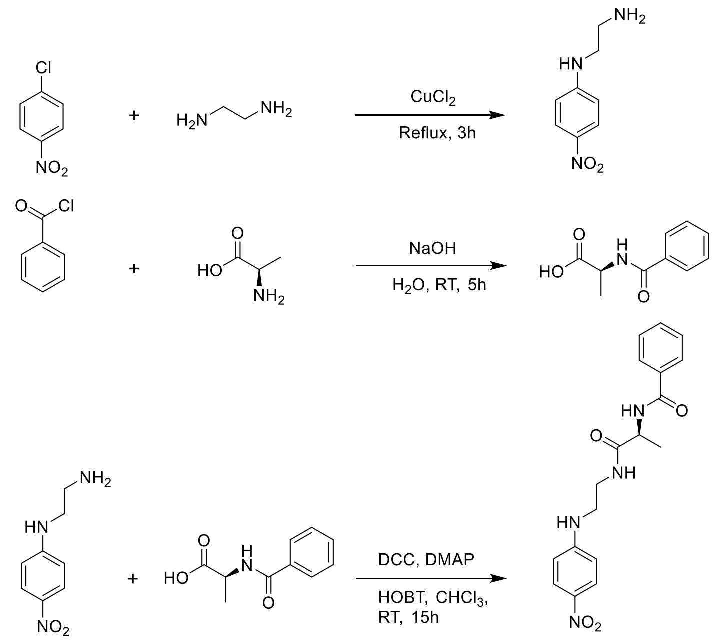

1a

1b

1

Synthetic Procedure for 1:

1a: In a 100 ml round bottomed flask, *p*-nitrochlorobenzene (24.0 g, 152.32 mmol) was added to a solution of anhydrous $CuCl_2$ (1.9 g, 14.07 mmol) in 42.8 mL (639.75 mmol) of ethylenediamine and mixture was refluxed for 3 hours under argon atmosphere. After completion of reaction the reaction mixture was cooled and 260 ml of water was added, stirred for 15 minutes, and filtered. Precipitate was recrystallized from boiling water and dried under vacuum to obtain product as orange solid: Yield- 48%. R_f = 0.52 (9:1 $CHCl_3$/MeOH). Melting point: 150˚C.

1b: 1b was synthesized following standard procedures reported elsewhere.[1]

For compound **1**, the following procedure was opted: In a 100 mL two necked round bottomed flask **1a** (4.50 g, 24.8 mmol) was taken with DMAP (3.79 g, 31.0 mmol) in 75.0 mL of dry chloroform and stirred for 15 minutes under inert atmosphere at 0◦C. Compound **1b** (3.2 g, 16.5 mmol) was added to this solution followed by subsequent addition of DCC (7.17 g, 35.0 mmol) and HOBT (2.8 g, 18.3 mmol) into ice cooled reaction mixture and continued the stirring for 15 h with gradual heating up to room temperature. Crude material was purified by column chromatography (silica, 100-200 mesh) using $CHCl_3$/MeOH as eluent. Yield: 45%. Melting point: 190 ◦C. ^{1}H NMR (500 MHz, DMSO-d_6, 300 K, TMS)**:** ™ = 8.55 (s, 1H, CO*NH*); 8.11 (br, m, 1H, CO*NH*); 7.99 (d, 2H, *Ar*); 7.91 (d, 2H, *Ar*); 7.54 (t, 1H, *Ar*); 7.47 (t, 2H, *Ar*); 7.31 (s, 1H, *NH*); 6.68 (d, 2H, *Ar*); 4.45 (m, 1H, *CH*); 3.26 (m, 4H, CH_2); 1.33 (d, 3H, CH_3). ^{13}C NMR (125 MHz, DMSO-d_6, 300 K, TMS): d = 173.26, 166.58, 154.97, 136.20, 134.44, 131.79, 128.62, 128.01, 126.73, 49.53, 42.21, 38.31, 18.31. Anal. Calcd. for $C_{18}H_{20}N_4O_4$: C 60.66, H 5.65, N 15.72; Found: C 60.78, H 5.53, N 15.83.

Results and Discussion

FT-IR Spectra of 1:

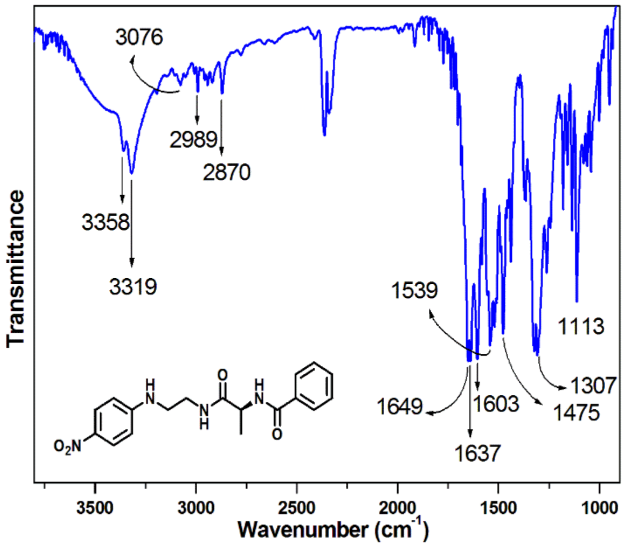

Figure S1. FT-IR Spectra of molecule 1-3.

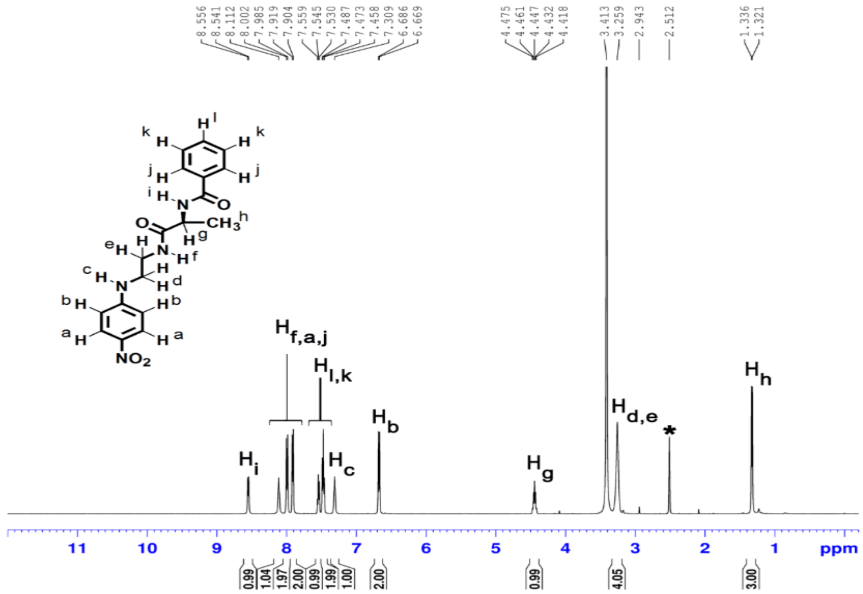

Figure S2. ^{1}H NMR Spectrum of Compound 1**:** 500 MHz ^{1}H NMR Spectrum of Compound **1** in DMSO-d_6, 300 K

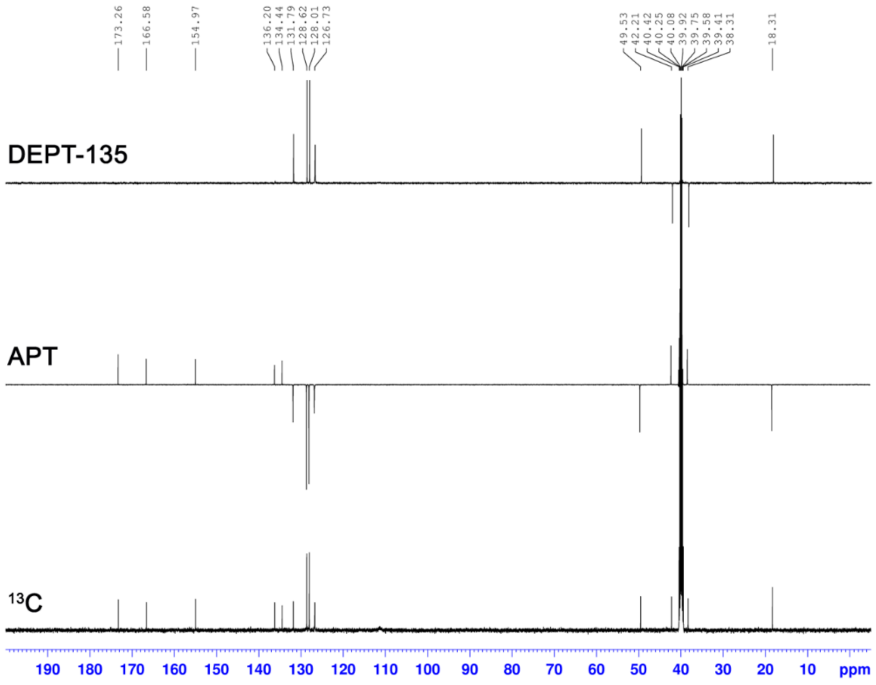

Figure S3. 125 MHz ^{13}C, APT and DEPT-135 NMR Spectra of Compound **1**.

.

Table S1. Crystal data and structure refinement for molecule **1**:

Identification code	**1**	
Empirical formula	C18 H20 N4 O4	
Formula weight	356.38	
Temperature	100(2) K	
Wavelength	0.71073 Å	
Crystal system	Triclinic	
Space group	P 1	
Unit cell dimensions	a = 5.189(5) Å	〈= 80.745(13)°.
b = 7.915(4) Å	®= 82.34(4)°.	
c = 10.643(3) Å	© = 88.94(5)°.	
Volume	427.6(5) Å3	
Z	1	
Density (calculated)	1.384 Mg/m3	
Absorption coefficient	0.100 mm^{-1}	
F(000)	188	
Crystal size	0.16 x 0.14 x 0.10 mm^3	
Theta range for data collection	1.96 to 25.00°.	
Index ranges	-6<=h<=5, -9<=k<=9, -12<=l<=12	
Reflections collected	6013	
Independent reflections	2601 [R(int) = 0.1161]	
Completeness to theta = 25.00°	98.5 %	
Absorption correction	Semi-empirical from equivalents	
Max. and min. transmission	0.74580 and 0.5093	
Refinement method	Full-matrix least-squares on F2	
Data / restraints / parameters	2601 / 3 / 236	
Goodness-of-fit on F2	1.017	
Final R indices [I>2sigma(I)]	R1 = 0.0815, wR2 = 0.2062	
R indices (all data)	R1 = 0.1421, wR2 = 0.2349	
Absolute structure parameter	-1(3)	
Largest diff. peak and hole	0.387 and -0.475 e.$Å^{-3}$	
CCDC No.	1861614	

Topography of thin film of molecule 1:

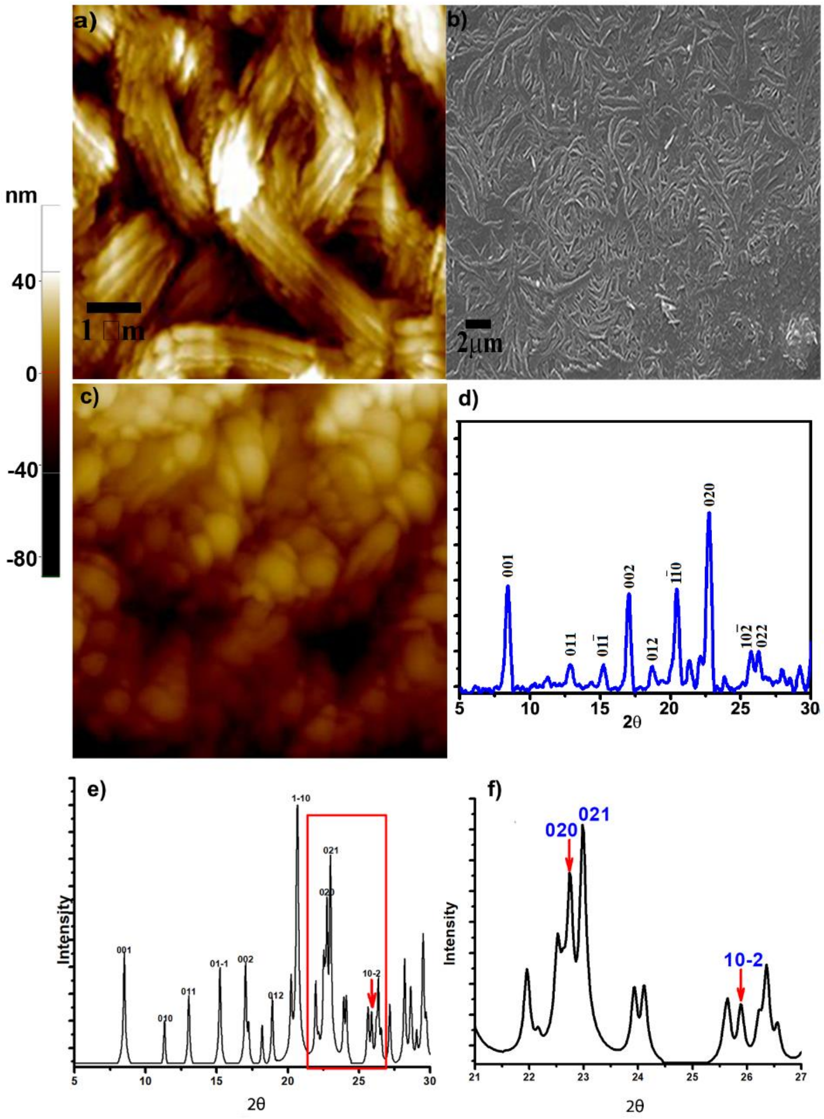

Figure S4: (a) AFM topographical image of thin film of **1** on ITO/glass substrate: scanned over 5×5 μm^2 area, (b) SEM image of the same thin film, (c) topography of crystalline thin film surface after thermal treatment of spin coated film at 120 °C for half an hour, (d) its powder XRD pattern, and (e), (f). Simulated powder XRD pattern from the cif.

Amplitude and Phase Plot of 1 by PFM Spectroscopy:

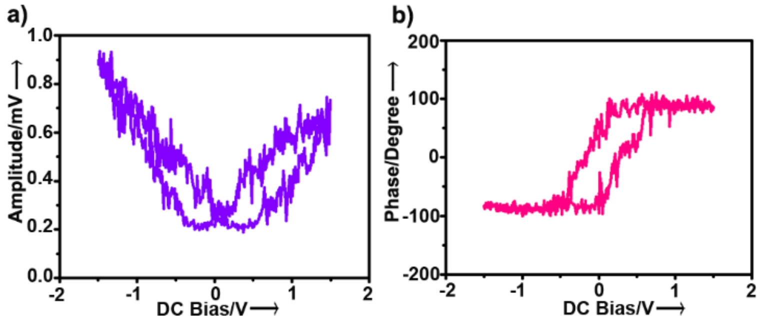

Figure S5: (a) Amplitude and (b) Phase hysteresis loops of **1** on thin film recorded by PFM as a function of DC bias voltage applied to the tip in the range of (+1.5 V) to (-1.5 V) with time period 1s.

Converse Piezocoefficient Calculation:

$$d_{33} = \frac{3\times \text{PFM amplitude (V)}}{\text{Detector(PSD) Sensitivity (V/m)} \times \text{Total voltage of PSD} \times \text{Tip Bias(AC, V)}} \text{.............(S1)}$$

where, amplitude corresponds to the maximum deformation value taken from the amplitude vs bias voltage plot; 3 is the instrument gain factor, ac voltage 3.5 V.

Table S5. Experimental and calculated data of crystal of **1**:

	Single Crystal XRD Data	Theoretically Optimized Data
Unit cell dimensions	a = 5.189(5) Å; α = 80.74°	a = 5.270 Å; α = 80.70°
	b = 7.915(4) Å; β = 82.34°	b = 7.875 Å; β = 82.13°
	c = 10.643(3) Å; γ = 88.94°	c = 10.614 Å; γ = 88.99°
Volume of unit cell	427.6(5) $Å^3$	430.6 $Å^3$
H-bond:		
-N=O····H-C- (r1)	2.351 Å	2.351 Å
=C-H····O=C- (r2)	2.352 Å	2.352 Å

Leakage Current measurement of 1:

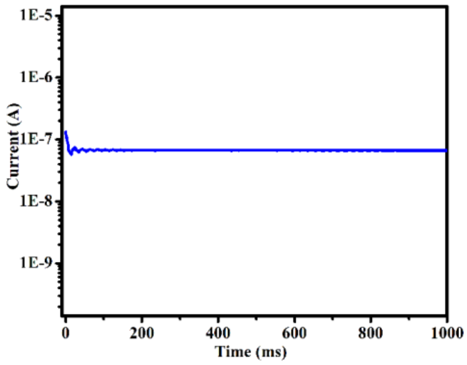

Figure S6. Current density (A) versus time (t) were recorded at the applied field of 20 kV/cm.

Plot for Modified Curie-Weiss Law at 100kHz frequency:

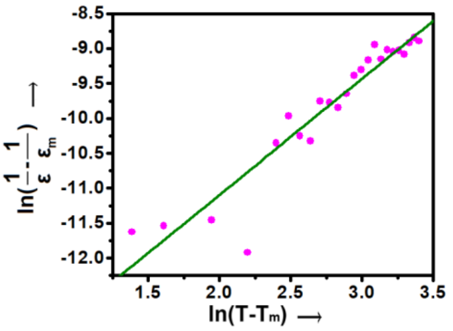

Figure S7. Modified Currie-Weiss law plot for variable temperature dielectric constant measurement at 100 kHz frequency

$$ {}^{1}/_{\varepsilon} - {}^{1}/_{\varepsilon_m} = {}^{(T-T_m)^{\gamma}}/_{C} \quad \text{..........(Eq. S1)} $$

where ε_m is the dielectric constant at transition temperature T_m, γ is diffusion coefficient having value from 1 to 2, and C is Curie constant. For normal ferroelectric material diffusion constant is less than 1.5. While diffusion constant is more than 1.5, the material is called as relaxor dielectric.

References

[1] V. V. Reddy, M. V. Naga, B. Rao, G. M. Reddy, K. Mukkanti and G. M. Reddy, *Synthetic Communications*, 2012, **42**, 3513-3523.
